# Ferritin as a key risk factor for nonalcoholic fatty liver disease in children with obesity

**DOI:** 10.1002/jcla.23602

**Published:** 2020-11-28

**Authors:** Junfeng Zhang, Jiajia Cao, Hui Xu, Guanping Dong, Ke Huang, Wei Wu, Jingjing Ye, Junfen Fu

**Affiliations:** ^1^ Department of Clinical Laboratory School of Medicine The Children’s Hospital Zhejiang University Zhejiang China; ^2^ National Clinical Research Center for Child Health Hangzhou China; ^3^ Department of Endocrinology School of Medicine The Children’s Hospital Zhejiang University Zhejiang China; ^4^ Department of Ultrasound School of Medicine The Children’s Hospital Zhejiang University Zhejiang China

**Keywords:** children, ferritin, nonalcoholic fatty liver disease, obesity, propensity score matching

## Abstract

**Background:**

The association between serum ferritin and nonalcoholic fatty liver disease (NAFLD) in children with obesity is not clear. This study was designed to investigate whether serum ferritin can be an independent predictor for NAFLD.

**Methods:**

According to the hepatic ultrasound results, a total of 347 children with obesity were enrolled in this study. Among them, 95 patients with NAFLD and 95 without NAFLD were matched for gender, age, blood pressure and body mass index, the odds ratios (OR) and 95% confidence intervals (CI) for the association of ferritin and the risk of NAFLD were analyzed.

**Results:**

After propensity score matching, ferritin values of the patients with NAFLD were significantly higher than those without NAFLD group. Alanine aminotransferase and ferritin were strongly associated with NAFLD in multivariate stepwise logistic regression analysis. The medium and high levels of ferritin increased risk of NAFLD, and the adjusted ORs were 3.298 (95% CI:1.326‐8.204), 7.322 (95% CI:2.725‐19.574) across the ferritin concentration tertiles after adjustment for confounders. Ferritin was shown to be the best predictor for NAFLD with sensitivity and specificity of 60.0% and 77.9%, respectively, area under the curve was 0.733.

**Conclusion:**

The results show that serum ferritin can usefully be considered as a predictor of NAFLD in children with obesity.

## Graphical abstract

If non‐invasive and simple biologic marker for NAFLD are found, it will be beneficial to children in early detection or screening for NAFLD. High levels of serum ferritin was found to be an increased risk of NAFLD. It can usefully be considered as a predictor of NAFLD in children with obesity

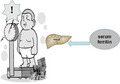

## 1 INTRODUCTION

Nonalcoholic fatty liver disease (NAFLD) is defined as hepatic steatosis in the absence of excessive alcohol consumption. It has emerged as the most common cause of chronic liver disease worldwide with estimated prevalence was 25% globally^1^ and approximately 21% in the Chinese population.^2^ NAFLD encompasses a spectrum of liver diseases ranging from nonalcoholic fatty liver to nonalcoholic steatohepatitis (NASH), which can progress to cirrhosis, hepatocellular carcinoma, and liver failure.^3^ It is increasingly challenging and become a major current health burden.^4^

According to the report, the prevalence of NAFLD in children is estimated to almost 3% in the general population and even 53% in obesity.^5^ It is expected with a rapidly growing in the future worldwide, due to the westernized diet and lifestyle. Therefore, the early diagnosis and proper treatment at appropriate time of NAFLD in children is becoming more and more important.

Liver biopsy is used for the diagnosis of patients with NAFLD as the gold standard method; however, it may be affected by other liver diseases during the image evaluation and limited by invasiveness.^6^ So, it is not feasible to recommend it in all patients with NAFLD, especially for children. There are several scoring systems have been investigated to diagnose NASH as well as fibrosis stage without histological data; however, the controversy associated with accuracy is ongoing.^7^ As a non‐invasive method, Fibroscan still cannot discriminate the entity of fibrosis because of its gray area and the tackle for magnetic resonance imaging is not widely used due to its high price and the exact cut‐off value has not been established.^8^, ^9^ Thus, researches are making efforts to seek cheap and non‐invasive biological markers for diagnosis and prognosis of NAFLD.

Recently, some investigations stated that serum ferritin are correlated with dyslipidemia,^10^ hypertension,^11^ central obesity,^12^ type 2 diabetes mellitus,^13^ liver disease,^14^ and stroke ^15^ in adults and also has a role in the development of metabolic syndrome in children and adolescents.^16^ It has reported that the level of serum ferritin can be an independent predictor of fibrosis in NAFLD patients based on its related to hepatic iron storage and hepatic inflammation^17^ and associated with insulin resistance and hepatocyte damage in NAFLD,^18^ while other researches have not made this findings,^19^ and there are few investigations assess the association between serum ferritin and NAFLD in children with obesity.

In this present study, we sought to evaluate the association between serum ferritin and NAFLD in children with obesity. It will be very helpful to provide medical treatment at an appropriate time if serum ferritin could be as a simple effective and less‐invasive biological marker, which reflect hepatic inflammatory change in obesity.

## 2 MATERIALS AND METHODS

### 2.1 Study population

Data from the Zhejiang university children's hospital from September 2018 to September 2019 were analyzed in the present study, a total of 347 children with obesity that met inclusion criteria for our final investigation, and participants were divided into two groups: with or without NAFLD. Obesity was defined as BMI for age and sex ≥95th percentile.^20^ All subjects has no iron deficiency or received iron food supplements or any drugs which could influence serum ferritin.

Nonalcoholic fatty liver disease was defined based on hepatic ultrasound which was conducted to assess the presence and extent of hepatic steatosis, according to the following guideline: (a) a diffuse hyperechoic texture (bright liver); (b) increased liver echo texture compared to kidney; (c) deep beam attenuation; (d) vascular blurring (absence of normal echogenic walls of the portal veins and hepatic veins).

The exclusion criteria included the following: (a) liver diseases such as alcohol consumption, drug induced hepatitis, viral hepatitis, autoimmune hepatitis, schistosomiasis, liver cirrhosis; (b) chronic diseases such as heart disease, kidney disease, epilepsy, tuberculosis, cancer, and other diseases which could affect liver function; (c) missing data on laboratory tests, ultrasound results, or physical examination.

All procedures performed in our study accordance with the guidelines of the Ethical Committee in our hospital and with the 1964 Helsinki Declaration and its later amendments.

### 2.2 Measurements

Height and waist circumference were measured to the nearest 0.1 cm, body weight nearest 0.1 kg. Body mass index (BMI) was calculated as weight(kg)/square of height(m^2^). A mercury sphygmomanometer was used to measure systolic blood pressure (SBP, mm Hg) and diastolic blood pressure (DBP, mm Hg) in the right upper arm after patients having rested for at least 10‐minute rest period. Blood pressure was assessed three times, at 2‐min intervals, and the mean of these three measurements was calculated.

### 2.3 Laboratory tests

Blood samples were collected after fasting overnight for at least 8 hours. All obtained blood samples were processed, refrigerated, transported to our clinical laboratory, and analyzed within 8 hours. All clinical analyses were performed by our hospital, a laboratory certified by the China National Accreditation Service for Conformity Assessment.

The serum concentrations of general biochemistry tests such as total cholesterol (TC), triglyceride (TG), high‐density lipoprotein cholesterol (HDL‐C), low‐density lipoprotein cholesterol (LDL‐C), uric acid, creatinine, ferritin, and liver chemistry such as total bilirubin, total protein, albumin, aspartate aminotransferase (AST), alanine transaminase (ALT), alkaline phosphatase(ALP), and gamma glutamyl transferase (GGT) were measured using a Analyzer AU5800 series (Beckman Coulter, USA). Examinations of hematologic testing were assessed by the BC‐5390 (Mindray, China), including white blood cell (WBC) counts, hemoglobin, and platelet counts. In addition, diabetes testing profile such as fasting plasma glucose (FPG), HbA1c, fasting insulin, and fasting C‐peptide were also measured using standard clinical laboratory techniques.

Insulin resistance (HOMA‐IR) was determined by the homeostasis model (16) and calculated using the following equation:

HOMA‐IR=(fastinginsulin[μIU/mL]×fastingglucose[mmol/L])/22.5

### 2.4 Statistical analyses

Normally distributed variables are presented as mean ± standard deviation (SD), while non‐normally distributed variables are presented as medians and interquartile range (25th–75th percentiles). Normally distributed variables were analyzed by independent sample t test, while non‐normally distributed variables were analyzed by the non‐parametric Mann‐Whitney *U* test.

Propensity analysis was carried out using logistic regression in order to create a propensity score for obese with NAFLD and without NAFLD. Propensity score matching analysis using a 1:1 greedy method without replacement. The caliper used in this study was 0.01, and the variables entered into the propensity model were gender, age, SBP, DBP, and BMI.

Analysis of stepwise logistic regression was conducted to determine the significant predictors after controlling for all variables. Statistical analyses were performed using SPSS 22.0 software, and a two‐sided *P*‐value < .05 was considered to be statistically significant.

## 3 RESULTS

### 3.1 Clinical characteristics of the study population

From 221 children with obesity were classified as NAFLD (mean age: 11.52 ± 2.39 years, 164 males and 57 females), 126 obese without NAFLD (mean age: 10.42 ± 2.62 years, 84 males and 42 females) based on ultrasound findings were selected for propensity matching. Finally, 95 patients were matched for gender, age, SBP, DBP, and BMI. The baseline clinical characteristics and laboratory data of the study population were described in Table 1. As expected, we found that ferritin, ALT, AST, GGT, ALP, total protein, LDL‐cholesterol, and insulin levels were significantly higher in obese with NAFLD than obese without NAFLD. No significant differences were observed for other variables between the two groups.

**Table 1:** Baseline characteristics of obese with or without NAFLD before and after propensity score matching

Parameters	Before matching		*P* value	After matching		*P* value
	Obese without NAFLD (n = 126)	Obese with NAFLD (n = 221)		Obese without NAFLD (n = 95)	Obese with NAFLD (n = 95)	
Gender (male/female)	84/42	164/57	.052	67/28	69/26	.749
Age (y)	10.42 ± 2.62	11.52 ± 2.39	<.001^***^	10.96 ± 2.46	10.80 ± 2.31	.644
Height (cm)	147.10 ± 15.54	154.73 ± 14.28	<.001^***^	151.07 ± 14.19	150.03 ± 12.85	.596
Weight (Kg)	60.62 ± 17.34	74.08 ± 19.31	<.001^***^	65.36 ± 16.16	64.27 ± 15.44	.636
BMI (kg/m^2^)	27.04 (24.44‐30.16)	29.69 (27.14‐32.80)	<.001^***^	27.76 (25.35‐30.63)	27.69 (26.25‐29.89)	.953
SBP (mm Hg)	121.02 ± 12.39	126.82 ± 13.42	<.001^***^	121.59 ± 13.00	121.92 ± 14.51	.87
DBP (mm Hg)	70.02 ± 9.60	72.25 ± 9.47	.037^*^	69.46 ± 9.41	69.28 ± 9.13	.894
Waist circumference (cm)	85.78 ± 9.96	94.80 ± 11.33	<.001^***^	88.72 ± 8.48	88.42 ± 7.65	.795
HB (g/L)	132.64 ± 9.79	134.70 ± 10.22	.067	132.47 ± 9.94	132.75 ± 8.96	.842
Platelet (10^9^/L)	319.65 ± 72.21	318.35 ± 69.98	.869	318.60 ± 73.72	318.30 ± 64.02	.976
WBC (10^9^/L)	8.81 ± 2.30	8.75 ± 1.87	.818	8.79 ± 2.16	8.78 ± 1.89	.96
Ferritin (μg/L)	39.80 (27.88‐57.63)	69.00 (46.93‐98.38)	<.001^***^	39.50 (27.80‐57.40)	63.90 (43.40‐94.20)	<.001^***^
Cholesterol (mmol/L)	3.36 (0.93‐4.14)	3.33 (1.36‐4.23)	.196	3.38 (1.10‐3.92)	3.27(1.34‐4.09)	.546
Triglyceride (mmol/L)	1.71 (0.98‐3.89)	2.12 (1.12‐3.87)	.304	1.60 (0.97‐3.64)	2.23(1.29‐4.18)	.056
HDL cholesterol (mmol/L)	1.16 (1.04‐1.35)	1.14 (0.98‐1.31)	.100	1.13 (1.01‐1.32)	1.18(1.03‐1.34)	.414
LDL cholesterol (mmol/L)	2.28 ± 0.60	2.36 ± 0.56	.218	2.20 ± 0.56	2.41 ± 0.60	.015^*^
Uric acid (μmol/L)	374.48 ± 94.19	422.03 ± 91.90	<.001^***^	382.73 ± 94.27	393.54 ± 80.59	.397
Creatinine (μmol/L)	60.00 (54.00‐65.00)	61.00 (56.00‐67.00)	.036^*^	61.00 (56.00‐66.00)	60.00 (55.00‐65.00)	.583
ALT (U/L)	18.00 (14.00‐26.00)	39.00 (22.00‐67.00)	<.001^***^	18.00 (14.00‐25.00)	40.00 (21.00‐59.00)	<.001^***^
AST (U/L)	22.00 (18.00‐27.00)	28.00 (22.00‐46.50)	<.001^***^	22.00 (18.00‐26.00)	28.00 (22.00‐41.00)	<.001^***^
GGT (U/L)	18.00 (15.00‐22.00)	27.00 (20.25‐37.75)	<.001^***^	18.00 (15.00‐22.00)	27.00 (19.00‐34.00)	<.001^***^
ALP (U/L)	275.14 ± 89.04	295.29 ± 103.91	.058	275.73 ± 94.73	309.62 ± 94.29	.014^*^
Total protein (g/L)	72.20 ± 5.94	73.72 ± 8.11	.066	71.90 ± 6.42	74.72 ± 5.35	.001^**^
Albumin (g/L)	42.56 ± 7.88	42.35 ± 9.01	.825	42.01 ± 8.85	42.34 ± 9.08	.800
Total bilirubin (μmol/L)	9.49 ± 4.18	10.18 ± 4.35	.152	9.44 ± 4.25	9.37 ± 4.08	.914
FBG (mmol/L)	5.90 (5.50‐6.60)	6.00 (5.40‐6.60)	.862	6.10 (5.50‐6.60)	6.00 (5.50‐6.50)	.998
HbA1c (%)	5.60 (5.38‐5.90)	5.70 (5.40‐5.90)	.310	5.60 (5.30‐5.90)	5.60 (5.40‐5.90)	.678
Fasting insulin (μIU/m/L)	18.85 (13.38‐27.10)	27.20 (17.13‐39.48)	<.001^***^	20.40 (14.30‐29.60)	22.90 (16.80‐36.70)	.037^*^
HOM‐IR	5.02 (3.38‐7.55)	7.38 (4.36‐11.29)	<.001^***^	5.55(3.66‐8.02)	6.42 (4.30‐10.20)	.053

Abbreviations: ALP, Alkaline phosphatase; ALT, Alanine aminotransferase; AST, Aspartate aminotransferase; BMI, body mass index; DBP, diastolic blood pressure; FBG, Fasting blood glucose; GGT, Gamma glutamyl transferase; HB, hemoglobin; HbA1c, Glycated hemoglobin; HOMA‐IR, insulin resistance by homoeostasis model; NAFLD, nonalcoholic fatty liver disease;SBP, systolic blood pressure; WBC, white blood cell.

*P < .05, ^**^ *P* < .01, ^***^ *P* < .001.

### 3.2 Logistic regression analysis for predictors of NAFLD

ALL the 29 variables were included in multivariate stepwise logistic regression analysis to determine independent predictors. As presented in Table 2, only ALT (OR: 1.066, 95% CI: 1.039‐1.093, *P* < .001) and ferritin (OR: 1.025, 95% CI: 1.012‐1.038, *P* < .001) were found to be an independent markers for the prediction of NAFLD.

**Table 2:** Risk factors of NAFLD from multivariate stepwise logistic regression

Parameters	Beta	Standard error	Odds ratio	95% CI	*P* value
Ferritin (μg/L)	0.025	0.007	1.025	1.012‐1.038	<.001
ALT (U/L)	0.064	0.013	1.066	1.039‐1.093	<.001

Abbreviations: ALT, Alanine aminotransferase; NAFLD, nonalcoholic fatty liver disease.

### 3.3 Associations between serum ferritin and NAFLD

All subjects were divided into three gradients based on ferritin tertiles through statistical software (1st Q: ferritin ≤ 39.0 μg/L; 2nd Q: ferritin was 39.1‐63.3 μg/L; and 3rd Q: ferritin ≥ 63.4 μg/L). We obtained another two models after controlling for gender, age, BMI, SBP, DBP, waist circumference (Model 1), model 1 plus ALT, uric acid, creatinine, triglyceride, HDL‐cholesterol, HbA1c, HOM‐IR, WBC counts, HB (Model 2), and using 1stQ (ferritin ≤ 39.0 μg/L) as references. It was observed that the medium and high levels of serum ferritin remained significantly independent associated with NAFLD in all models. The results were shown in Table 3.

**Table 3:** Associations between serum Ferritin and NAFLD

Ferritin (μg/L)	Case,n(%)	unadjusted		Model 1^a^		Model 2^b^	
		Odds ratio (95% CI)	*P 1* value	Odds ratio (95% CI)	*P 2*value	Odds ratio (95% CI)	*P* 3value
≤39.0	65 (34.2)	1.00		1.00		1.00	
39.1‐63.3	62 (32.6)	2.295 (1.097‐4.798)	<.001	2.299 (1.060‐4.984)	.035	3.298 (1.326‐8.204)	.010
≥63.4	63 (33.2)	8.356 (3.775‐18.494)	<.001	9.752 (4.211‐22.532)	<.001	7.322 (2.725‐19.574)	.011

Abbreviation: NAFLD: nonalcoholic fatty liver disease.

^a^ Model 1, adjusted for gender, age, BMI, SBP, DBP, waist circumference.

^b^ Model 2, adjusted for gender, age, BMI, SBP, DBP, waist circumference, ALT, uric acid, creatinine, triglyceride, HDL‐cholesterol, HbA1c, HOM‐IR, WBC, HB.

**Figure 1:**
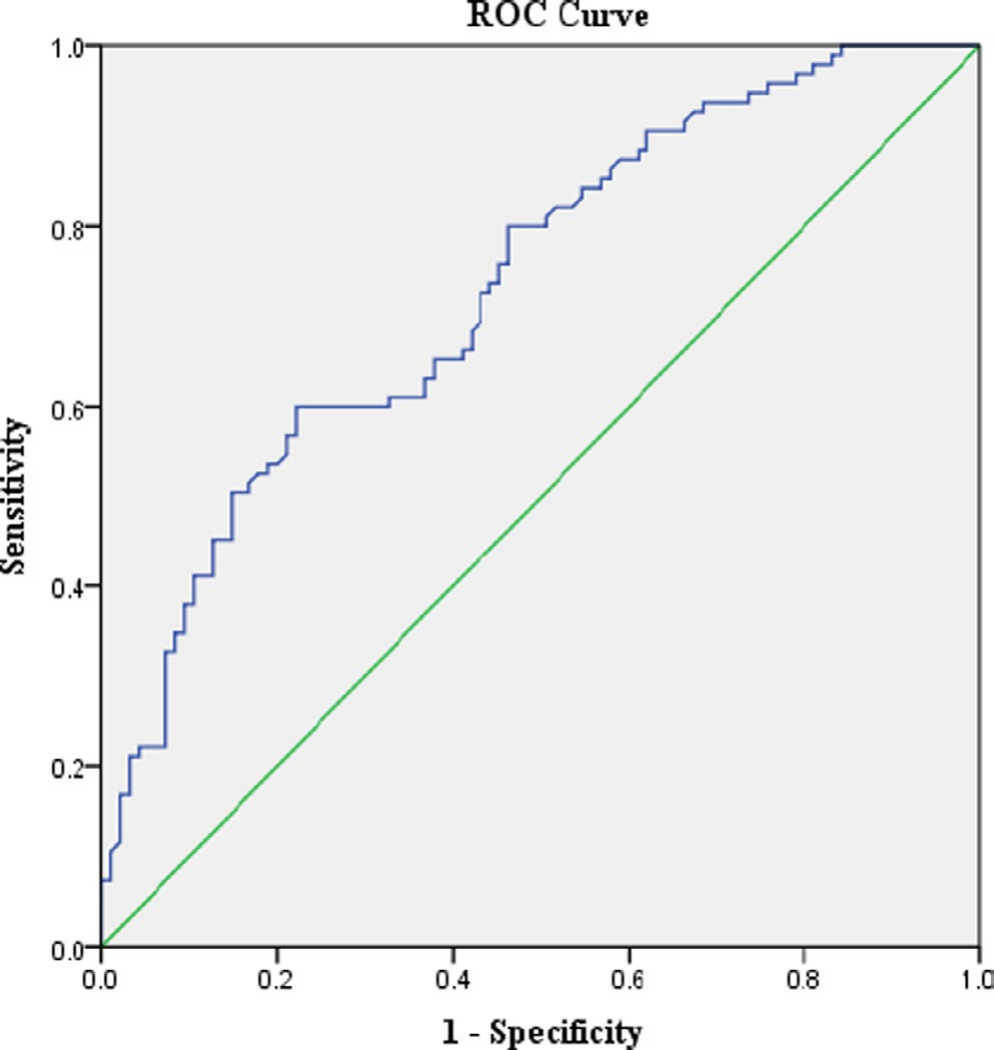
Receiver operating characteristic curve of serum ferritin to predict the occurrence of NAFLD in children with obesity

The receiver operating characteristic curve analysis revealed the relationship between serum ferritin and NAFLD, and the area under the curve was 0.733 (95% CI: 0.663‐0.803; *P* < .001). Using the best cut‐off value of ferritin predicted, the occurrence of NAFLD was ≥ 58.55 μg/L with a sensitivity of 60.0% and specificity of 77.9% (Figure 1).

## 4 DISCUSSION

This study focused on the association between ferritin and NAFLD in children with obesity by using a propensity score method. We observed that NAFLD patients had higher ferritin levels than control subjects, and elevated serum ferritin level was associated with a significantly increased risk of NAFLD.

Hsiao TJ^21^ reported that iron metabolism disorders can be related to the development of NAFLD. Jeong DW^22^ demonstrated that the level of serum ferritin was a better marker than vitamin D for predicting NAFLD in Korean adults. Although several studies showed that ferritin cannot indicate the stage of NAFLD,^23^, ^24^ the number of studies indicated that elevated serum ferritin was an independent risk factor associated with NAFLD in US,^25^ Japanese^26^ and Chinese adults.^27^, ^28^

Three‐hit hypothesis is well‐known NAFLD pathogenesis that takes the progression to NASH and fibrosis depends on additional factors such as oxidative stress, inflammatory cytokines, free fatty acids, and mitochondrial dysfunction in the base of obesity.^5^ Serum ferritin is the main iron‐storage protein in the liver, where the majority of extra body iron is stored. Some studies have shown that elevated deposition of hepatic iron was an important factor in catalyzing reactive oxygen species generation, which is considered to be the second hit. The production of reactive oxygen species involved in increase of oxidative stress damage to cells and tissue may result in insulin resistance,^29^, ^30^ which plays a key role in NAFLD development.^31^

Ferritin levels can be increased secondary due to viral hepatitis, steatohepatitis, chronic consumption of alcohol as well as obesity.^17^ Hyperferritinemia observed in obesity‐related chronic inflammation such as metabolic syndrome, diabetes mellitus, and NAFLD,^17^ and obesity led to hyperferritinemia are considered as due to inflammation responsible for high levels of ferritin.^32^ Several studies demonstrated that higher levels of ferritin are considered as marker of hepatic damage, because of inflammatory cytokine activation.^33^, ^34^ It has been reported that hepatic iron accumulation can stimulate production of inflammatory cytokines which is significantly related to hepatic fibrosis in NAFLD patients.^35^, ^36^ According to the research by Kowdley ^17^who found that ferritin has a significant association with the histological features of NAFLD including hepatocellular ballooning, steatosis and fibrosis.

A number of studies have found that ALT values were higher in patients with NAFLD. A study among 25,597 Korean participants demonstrated that elevation of serum ferritin was positively associated with NAFLD and ALT elevation,^37^ and the findings were similar with You G,^28^ who observed normal weight Chinese adults in a cross‐sectional study. In addition, Dubern B^38^ reported that higher ferritin levels were significantly associated with elevation of ALT in severely obese children.

In the present study, we evaluated the associations of serum ferritin with the risk of NAFLD and elevated ALT in children with obesity. After matching the potential confounders such as gender, age, SBP, DBP, and BMI to balance these baseline characteristics. Through multivariate stepwise regression analysis, we found that elevated serum ferritin has a significant association with increased risk of NAFLD.

Nonetheless, it has several limitations in this study. First, the sample of subjects is relatively small. Second, the diagnosis of NAFLD is according to the results of hepatic ultrasound in this study although liver biopsy is still as the gold standard method. Furthermore, there are other factors such as race/ethnicity, lifestyle‐related parameters, dietary intake of nutrients need to be further addressed in future studies.

In conclusion, we concluded that elevated serum ferritin can usefully be considered as a predictor of NAFLD in children with obesity.

## CONFLICT OF INTERESTS

The authors have no conflict of interests for this paper.

## AUTHOR CONTRIBUTIONS

Jun‐Feng Zhang involved in conceptualization, writing‐original draft preparation, and writing‐reviewing and editing. Jia‐Jia Cao involved in investigation, formal analysis, and data curation. Hui Xu, Guan‐Ping Dong, Ke Huang, Wei Wu, and Jing‐Jing Ye involved in investigation. Jun‐Fen Fu involved in supervision, conceptualization, and writing‐reviewing and editing. All authors read and approved the final version.

## INFORMED CONSENT

Informed consent was obtained from the parents or legal guardians of all patients.
